# Associations between physical activity participation and different types of sports consumption expenditures among older adults: a cross-sectional study

**DOI:** 10.3389/fpubh.2025.1713320

**Published:** 2025-12-17

**Authors:** Chong Zhou, Qing Ji, Xiao Shi, Heng Liu, Yuanxin Chen

**Affiliations:** 1 Post-doctoral Station, Central China Normal University, Wuhan, Hubei, China; 2 School of Physical Education, Central China Normal University, Wuhan, Hubei, China; 3 School of Physical Education, China University of Geosciences, Wuhan, Hubei, China

**Keywords:** healthy aging, older adults, physical activity, sport consumption, physical activity participation, preventive health, public health policy

## Abstract

**Background:**

As populations age, promoting physical activity among older adults becomes increasingly critical for healthy aging. Sport consumption, covering spending on products, services, and rehabilitation, may play a key role in shaping exercise behaviors, but its influence remains underexamined.

**Methods:**

A cross-sectional survey of 395 older adults in Wuhan, China measured expenditures in three sport consumption categories and analyzed their associations with exercise frequency, duration, and perceived intensity. Socioeconomic variables were also considered.

**Results:**

Greater sport consumption was significantly associated with higher levels of physical activity. Among respondents, 49.4% exercised 3–7 times per week, and 51.9% reported high-intensity activity. Those spending over 1,000 RMB annually on sports products or rehabilitation services were more likely to engage in frequent and vigorous exercise. Income and education positively correlated with consumption levels; however, 60.5% of respondents spent ≤500 RMB on sports products, and 89.4% on participation services.

**Conclusion:**

Sport-related spending reflects not only financial capacity but also a proactive health orientation. Promoting active aging requires policies that enhance accessibility through public facilities, subsidies, and health education tailored to older adults, especially those with lower income and education levels.

## Introduction

Population aging is one of the most defining demographic transitions of the 21st century, posing profound challenges to global public health systems, social security frameworks, and economic sustainability (1–3). According to the United Nations Department of Economic and Social Affairs (UNDESA), the global population aged 60 years and older is projected to reach 1.42 billion by the end of 2025, with its share of the total population continuing to rise (4). As the number and proportion of older adults increase, the pursuit of “healthy aging” has become a key concern for governments and international organizations worldwide.

In 2020, the World Health Organization (WHO) launched the global initiative “Decade of Healthy Ageing 2021–2030,” which advocates a life-course approach to aging and calls for the development of supportive systems that help older adults maintain functional ability and improve quality of life. The initiative outlines four key areas for action: changing how we think, feel, and act toward age and aging; fostering communities that support the abilities of older people; delivering person-centered, integrated care and primary health services responsive to older people; and providing access to long-term care for older adults who need it (5). This framework establishes a functional and participation-centered understanding of aging that emphasizes the interconnections between physical independence, mental well-being, and social engagement. Globally, many countries have introduced targeted policies and interventions to promote the goals of healthy aging. For instance, Nordic countries have embraced the concept of “life-course health” (6), integrating physical activity and social participation into strategies to delay aging; Japan has promoted “Smart Wellness Cities” that encourage walkability and interpersonal interaction for older adults through optimized urban design (7); and Germany has implemented the “Movement and Health in Old Age” program (8), which combines community-level exercise interventions with health education. In China, the challenge of population aging is particularly acute. According to the National Bureau of Statistics, by the end of 2024, the population aged 60 and over reached 310 million, accounting for 22.0% of the total population (9). To actively respond to this demographic shift, the Chinese government has advanced the “Healthy China Initiative,” integrating older adult physical fitness into both the national fitness and public health systems. Policies such as the “14th Five-Year Plan for Healthy Aging” and the “14th Five-Year National Fitness Plan” emphasize the development of sports services for the older adults, the construction of age-friendly public spaces, and the encouragement of physical activity among older populations. As an effective means of enhancing physical functioning, psychological health, and social integration (10–12), physical activity has increasingly become a central pillar in global strategies aimed at healthy aging. Regular physical activity yields a broad spectrum of health benefits for older adults, encompassing physical, psychological, and social dimensions (13, 14). Physiologically, exercise improves cardiovascular function, enhances musculoskeletal strength, slows the progression of sarcopenia and osteoporosis, and reduces the risk of chronic conditions such as diabetes, hypertension, and stroke (15). Psychologically, physical activity has been linked to improved cognitive functioning, lower rates of depression and anxiety, and enhanced overall mood (16). Moreover, physical activity often occurs in social settings, such as group exercises or community walking programs, offering older adults opportunities for social engagement, reducing loneliness and fostering a sense of belonging (17). Thus, from a public health perspective, promoting physical activity among older adults is a highly cost-effective intervention with multidimensional returns.

While the health benefits of physical activity are well-established, there is increasing recognition that participation in sport and exercise is often mediated by consumption behavior. For older adults, sports-related consumption may include expenditure on fitness equipment, sports apparel, gym or membership fees, rehabilitation services, and wellness supplements. These expenditures are not merely economic transactions, they represent investments in physical capacity, self-care, and functional independence. In this regard, sports consumption can be conceptualized as a form of “preventive health spending,” where individuals allocate financial resources to maintain or enhance health status in later life (18). However, disparities in economic capital, educational background, and health literacy may lead to unequal patterns of sports consumption and, by extension, unequal access to the benefits of physical activity (19–21). Existing studies have extensively explored the psychosocial or environmental determinants of older adults’ physical activity (22–25), yet relatively few have examined how different levels or types of sports consumption expenditure relate to participation behavior. In particular, there is a lack of empirical evidence analyzing older adults’ physical activity through the lens of financial investment in health-related consumption. Given the growing diversity in older adults’ consumption patterns and health behaviors, this perspective deserves more academic attention.

This study aims to investigate the association between sports consumption expenditure and physical activity participation among older adults. Using data from a cross-sectional questionnaire survey conducted in Wuhan, China, among 395 individuals aged 60 and above. This classification aims to reflect the multifaceted nature of older adult sport consumption and explore its association with physical activity behaviors. Crosstab analysis is used to describe how different expenditure levels correspond to variations in exercise frequency, duration, and intensity. By doing so, the study provides foundational data for designing differentiated public health interventions and supporting policies on active aging.

## Methods

### Data sources

Data were collected using a structured questionnaire designed specifically for this study. The questionnaire included three sections: (1) sociodemographic characteristics (e.g., sex, education, income); (2) physical activity participation (e.g., frequency, duration, intensity); and (3) sport-related consumption expenditures (e.g., spending on sports products, participation services, and rehabilitation). The full English version of the questionnaire is provided in Supplementary File S1.

A cross-sectional questionnaire survey was conducted in Wuhan, China, targeting individuals aged 60 years and above. A stratified random sampling approach was adopted based on age groups to ensure representativeness of the older adult population. To further ensure diversity within the sampling frame, the survey covered ten major urban districts of Wuhan, including Hanyang District, Jianghan District, Jiang’ an District, Qiaokou District, Wuchang District, Wuhan Economic and Technological Development Zone (Wuhan ETDZ), Qingshan District, Donghu High-tech Development Zone, Hongshan District, and the East Lake Scenic Area. These districts vary in socioeconomic status, population density, and spatial characteristics, providing a heterogeneous sample of older adults living in Wuhan’s urban communities. It should be noted that the sample represents the older adult population residing in Wuhan’s urban areas and is not intended to generalize to the national urban older adult population. Trained investigators administered the survey face-to-face using paper-based questionnaires. For respondents with low literacy or no formal education, an interview-based method was employed in which investigators read each item aloud and recorded the answers on their behalf. A total of 400 questionnaires were distributed, and 395 were deemed valid after data cleaning and quality checks. All participants provided informed consent prior to participation.

### Measures

#### Sport consumption expenditure

Sport-related expenditure was categorized into three domains: sports product consumption, which includes spending on tangible sports products such as sportswear, shoes, hats, and equipment. Respondents were asked: *“How much did you spend on physical sports goods in the past year?”* with responses coded as: (1 = ≤500 RMB, 2 = 501–1,000 RMB, 3 = 1,001–2000 RMB, 4 = 2001–3,000 RMB, 5 = > 3,000 RMB). Participating sports consumption, referring to expenditures related to participation in sports activities, including coaching fees, gym or club memberships, venue rentals, and ticket purchases. This was assessed by the question: *“How much did you spend on participatory sports services in the past year?*,” using the same five-point scale (1 = ≤500 RMB, 2 = 501–1,000 RMB, 3 = 1,001–2000 RMB, 4 = 2001–3,000 RMB, 5 = > 3,000 RMB). Consumption of exercise rehabilitation services, covering costs for exercise prescriptions, physical rehabilitation services, and nutritional supplements. Respondents were asked: *“How much did you spend on rehabilitative sports services in the past year?*,” also using the same response options (1 = ≤500 RMB, 2 = 501–1,000 RMB, 3 = 1,001–2000 RMB, 4 = 2001–3,000 RMB, 5 = > 3,000 RMB).

#### Sport participation behavior

Three indicators were used to measure physical activity behavior: Exercise frequency was measured by the question: *“How often do you engage in physical fitness activities each week?*,” with responses coded as: (1 = 1–2 times/week, 2 = 3–7 times/week, 3 = more than 7 times/week). Exercise duration per session was assessed with the item: “How long do your exercise sessions usually last?” (1 = ≤60 min, 2 = 61–120 min, 3 = > 120 min). Perceived exercise intensity was measured via the question: “How would you describe the intensity of your typical exercise session?” (1 = Low: no significant change in breathing or heart rate, 2 = Moderate: slightly faster breathing and light sweating, 3 = High: rapid breathing, noticeably increased heart rate, and heavy sweating).

#### Sociodemographic characteristics

The following variables were included as covariates in the analysis: Sex (0 = male, 1 = female). Education level (1 = primary school or below, 2 = junior high school, 3 = high school/technical/vocational, 4 = bachelor’s degree or above). Monthly individual income (pre-tax) (1 = ≤4,000 RMB, 2 = 4,001–6,000 RMB, 3 = > 6,000 RMB). Annual household income (1 = ≤40,000 RMB, 2 = 40,001–80,000 RMB, 3 = > 80,000 RMB).

These variables were used to control for potential confounding effects in the multivariate models examining the association between physical activity and sport-related consumption patterns.

### Statistical analysis

This study employed SPSS 27.0 software to conduct statistical analysis of the survey data. Descriptive statistics were performed on demographic characteristics, physical activity participation indicators, and sport consumption-related variables. The analysis examined the associations between participants’ gender, education level, monthly personal income, and annual household income with their sport consumption patterns. In addition, descriptive analyses were conducted to explore the characteristics of participants’ physical activity engagement and their corresponding sport consumption behavior.

## Results

### Sociodemographic and physical activity characteristics of the respondents

Table 1 summarizes the demographic characteristics and exercise-related behaviors of the 395 valid respondents. The gender distribution was nearly equal, with 50.6% male and 49.4% female. Regarding education level, 44.3% had completed senior high school, 25.8% had junior high school education, 19.7% had a bachelor’s degree or above, and 10.1% had only primary school education or below.

**Table 1:** The descriptive statistics of valid sample characteristics.

Variables	Categories	*N* (395)	%
Sex	Male	200	50.6
	Female	195	49.4
Education level	Primary school or below	40	10.1
	Junior high school	102	25.8
	Senior high school	175	44.3
	Bachelor’s degree or above	78	19.7
Monthly individual income (pre-tax)	≤4,000 RMB	288	72.9
	4,001–6,000 RMB	70	17.7
	>6,000 RMB	37	9.4
Annual household income	≤40,000 RMB	174	44.1
	40,001–80,000 RMB	147	37.2
	>80,000 RMB	74	18.7
Exercise frequency	1–2 times/week,	124	31.4
	3–7 times/week,	195	49.4
	more than 7 times/week	76	19.2
Exercise duration per session	≤60 min	316	80.0
	61–120 min	51	12.9
	>120 min	28	7.1
Perceived exercise intensity	Low	126	31.9
	Moderate	64	16.2
	High	205	51.9
Sports product consumption	≤500 RMB	239	60.5
	501–1,000 RMB	88	22.3
	1,001–2000 RMB	49	12.4
	2001–3,000 RMB	11	2.8
	>3,000 RMB	8	2.0
Participating sports consumption	≤500 RMB	353	89.4
	501–1,000 RMB	35	8.9
	1,001–2000 RMB	5	1.3
	2001–3,000 RMB	1	0.3
	>3,000 RMB	1	0.3
Consumption of exercise rehabilitation services	≤500 RMB	322	81.5
	501–1,000 RMB	46	11.6
	1,001–2000 RMB	13	3.3
	2001–3,000 RMB	3	0.8
	>3,000 RMB	11	2.8

Low: no significant change in breathing or heart rate; Moderate: slightly faster breathing and light sweating; High: rapid breathing, noticeably increased heart rate, and heavy sweating.

In terms of monthly individual income, the majority (72.9%) earned 4,000 RMB or less, while 17.7% earned between 4,001 and 6,000 RMB, and 9.4% earned more than 6,000 RMB. For annual household income, 44.1% reported 40,000 RMB or less, 37.2% reported between 40,001 and 80,000 RMB, and 18.7% reported more than 80,000 RMB.

With regard to physical activity behaviors, 49.4% of respondents exercised 3–7 times per week, 31.4% exercised 1–2 times, and 19.2% reported more than 7 sessions weekly. Most participants exercised for 60 min or less per session (80.0%), while 12.9% exercised for 61–120 min, and only 7.1% exercised for over 120 min. In terms of perceived exercise intensity, 51.9% reported high intensity, 16.2% moderate, and 31.9% low intensity.

Concerning sports consumption, 60.5% of respondents spent 500 RMB or less annually on sports products, and 89.4% spent 500 RMB or less on participation-related sports consumption (e.g., membership fees, coaching, or event tickets). In terms of exercise rehabilitation services, 81.5% spent 500 RMB or less, and only a small proportion spent over 1,000 RMB.

### Cross-tabulation of demographic characteristics and types of sport consumption

Table 2 presents the cross-tabulation results of sex and education level with three categories of sport consumption: sports product consumption, participating sports consumption, and exercise rehabilitation services consumption.

**Table 2:** Distribution of sport consumption expenditures by demographic characteristics: sex and education level.

Variables	Categories	*N* (395)	Sports product consumption					Participating sports consumption					Consumption of exercise rehabilitation services				
			1	2	3	4	5	1	2	3	4	5	1	2	3	4	5
Sex	Male (%)	200	122 (61.00)	38 (19.00)	27 (13.50)	9 (4.50)	4 (2.00)	177 (88.50)	17 (8.50)	4 (2.00)	1 (0.50)	1 (0.50)	164 (82.00)	25 (12.50)	7 (3.50)	1 (0.50)	3 (1.50)
	Female (%)	195	117 (60.00)	50 (25.64)	22 (11.28)	2 (1.03)	4 (2.05)	176 (90.26)	18 (9.23)	1 (0.51)	0 (0.00)	0 (0.00)	158 (81.03)	21 (10.77)	6 (3.08)	2 (1.03)	8 (4.10)
Education level	Primary school or below (%)	40	23 (57.50)	13 (32.50)	2 (5.00)	2 (5.00)	0 (0.00)	36 (90.00)	4 (10.00)	0 (0.00)	0 (0.00)	0 (0.00)	33 (82.50)	5 (12.50)	1 (2.50)	1 (2.50)	0 (0.00)
	Junior high school (%)	102	53 (51.96)	21 (20.59)	22 (21.57)	4 (3.92)	2 (1.96)	93 (91.18)	8 (7.84)	1 (0.98)	0 (0.00)	0 (0.00)	82 (80.39)	11 (10.78)	5 (4.90)	0 (0.00)	4 (3.92)
	Senior high school (%)	175	111 (63.43)	40 (22.86)	17 (9.71)	3 (1.71)	4 (2.29)	157 (89.71)	13 (7.43)	4 (2.29)	0 (0.00)	1 (0.57)	144 (82.29)	24 (13.71)	5 (2.86)	0 (0.00)	2 (1.14)
	bachelor’s degree or above (%)	78	52 (66.67)	14 (17.95)	8 (10.26)	2 (2.56)	2 (2.56)	67 (85.90)	10 (12.82)	0 (0.00)	1 (1.28)	0 (0.00)	63 (80.77)	6 (7.69)	2 (2.56)	2 (2.56)	5 (6.41)

1 = ≤500 RMB; 2 = 501 ~ 1,000 RMB; 3 = 1,001 ~ 2000 RMB; 4 = 2001 ~ 3,000 RMB; 5 = > 3,000 RMB.

In terms of sex, both male and female respondents showed similar patterns in sports product consumption, with the majority spending ≤500 RMB (61.0% for males and 60.0% for females). Males were slightly more likely to report moderate-to-high consumption levels (≥1,001 RMB), especially in the consumption of sports products and exercise rehabilitation services. Regarding participating sports consumption, over 88% of both sexes reported expenditures ≤500 RMB. For exercise rehabilitation services, 82.0% of males and 81.0% of females reported expenditures ≤500 RMB, with females showing a slightly higher proportion in the highest category (>3,000 RMB, 4.1% vs. 1.5%).

With regard to education level, individuals with higher education levels tended to spend more across all three categories. Among those with a bachelor’s degree or above, 66.7% spent ≤500 RMB on sports products, and 17.9% spent between 501–1,000 RMB, with 15.4% spending over 1,000 RMB. In contrast, those with primary school education or below had over 90% of respondents concentrated in the lowest spending category (≤500 RMB) across all three consumption types. Participation and rehabilitation-related expenditures were especially low among less-educated individuals.

### Cross-tabulation of income with sport consumption categories

Table 3 presents the cross-tabulation results of individual monthly income and household annual income with three categories of sport consumption: sports product consumption, participating sports consumption, and exercise rehabilitation services consumption.

**Table 3:** Distribution of sport consumption expenditures by economic characteristics: monthly individual income and annual household income.

Variables	Categories	*N* (395)	Sports product consumption					Participating sports consumption					Consumption of exercise rehabilitation services				
			1	2	3	4	5	1	2	3	4	5	1	2	3	4	5
Monthly individual income	≤4,000 RMB (%)	288	176 (61.11)	65 (22.57)	32 (11.11)	8 (2.78)	7 (2.43)	259 (89.93)	24 (8.33)	3 (1.04)	1 (0.35)	1 (0.35)	238 (82.64)	29 (10.07)	10 (3.47)	2 (0.69)	9 (3.13)
	4,001 ~ 6,000 RMB (%)	70	38 (54.29)	18 (25.71)	11 (15.71)	2 (2.86)	1 (1.43)	60 (85.71)	8 (11.43)	2 (2.86)	0 (0.00)	0 (0.00)	52 (74.29)	15 (21.43)	2 (2.86)	1 (1.43)	0 (0.00)
	>6,000 RMB (%)	37	25 (67.57)	5 (13.51)	6 (16.22)	1 (2.70)	0 (0.00)	34 (91.89)	3 (8.11)	0 (0.00)	0 (0.00)	0 (0.00)	32 (86.49)	2 (5.41)	1 (2.70)	0 (0.00)	2 (5.41)
Annual household income	≤40,000 RMB (%)	174	101 (58.05)	43 (24.71)	20 (11.49)	4 (2.30)	6 (3.45)	152 (87.36)	18 (10.34)	4 (2.30)	0 (0.00)	0 (0.00)	135 (77.59)	21 (12.07)	9 (5.17)	1 (0.57)	8 (4.60)
	40,001 ~ 80,000 RMB (%)	147	87 (59.18)	34 (23.13)	21 (14.29)	3 (2.04)	2 (1.36)	130 (88.44)	14 (9.52)	1 (0.68)	1 (0.68)	1 (0.68)	125 (85.03)	17 (11.56)	2 (1.36)	2 (1.36)	1 (0.68)
	>80,000 RMB (%)	74	51 (68.92)	11 (14.86)	8 (10.81)	4 (5.41)	0 (0.00)	71 (95.95)	3 (4.05)	0 (0.00)	0 (0.00)	0 (0.00)	62 (83.78)	8 (10.81)	2 (2.70)	0 (0.00)	2 (2.70)

1 = ≤500 RMB; 2 = 501 ~ 1,000 RMB; 3 = 1,001 ~ 2000 RMB; 4 = 2001 ~ 3,000 RMB; 5 = > 3,000 RMB.

In terms of individual monthly income, a clear income gradient was observed across all consumption categories. Among respondents earning ≤4,000 RMB per month, a substantial majority reported low levels of consumption, with 61.1% spending ≤500 RMB on sports products, 89.9% on participating sports, and 82.6% on rehabilitation services. Moderate-to-high consumption levels (≥1,001 RMB) were rare in this group. As income increased to 4,001–6,000 RMB, the proportion of individuals reporting mid-level consumption (501–2000 RMB) rose modestly across all categories. For example, 15.7% spent 1,001–2000 RMB on sports products, and 21.4% did so for rehabilitation services. Among respondents with monthly income >6,000 RMB, higher expenditure patterns became more pronounced. Specifically, 16.2% spent 1,001–2000 RMB on sports products, and 8.1% spent ≥1,001 RMB on rehabilitation services, suggesting a positive association between income level and consumption willingness.

With regard to household annual income, similar patterns were observed. In households earning ≤40,000 RMB annually, the majority of respondents spent ≤500 RMB on all three consumption types (58.1% for sports products, 87.4% for participation, and 77.6% for rehabilitation). In contrast, households with annual income >80,000 RMB showed a relatively higher tendency toward moderate-to-high spending. For instance, 10.8% reported spending 1,001–2000 RMB on sports products, and 5.4% spent over 2000 RMB on rehabilitation services. The proportion of respondents in the lowest spending bracket decreased notably with increasing income.

Overall, both monthly individual income and annual household income were positively associated with sport consumption levels. Higher-income individuals and families tended to spend more across all three categories, particularly in exercise rehabilitation services, which showed the greatest disparity across income levels.

### Cross-tabulation of exercise behavior with sport consumption categories

Table 4 presents the cross-tabulation results of three dimensions of exercise behavior: exercise frequency, exercise duration per session, and perceived exercise intensity, with three categories of sport consumption: sports product consumption, participating sports consumption, and exercise rehabilitation services consumption.

**Table 4:** Distribution of sport consumption expenditures by exercise-related characteristics: exercise frequency, exercise duration per session, and perceived exercise intensity.

Variables	Categories	*N* (395)	Exercise frequency			Exercise duration per session			Perceived exercise intensity		
			1 ~ 2 times/week	3 ~ 7 times/week	More than 7 times/week	≤60 minutes	61 ~ 120 minutes	>120 minutes	Low	Moderate	High
Sports product consumption	≤500 RMB (%)	239	71 (29.71)	120 (50.21)	48 (20.08)	190 (79.50)	28 (11.72)	21 (8.79)	72 (30.13)	42 (17.57)	125 (52.30)
	501 ~ 1,000 RMB (%)	88	28 (31.82)	46 (52.27)	14 (15.91)	69 (78.41)	15 (17.05)	4 (4.55)	27 (30.68)	13 (14.77)	48 (54.55)
	1,001 ~ 2000 RMB (%)	49	19 (38.78)	22 (44.90)	8 (16.33)	41 (83.67)	6 (12.24)	2 (4.08)	19 (38.78)	8 (16.33)	22 (44.90)
	2001 ~ 3,000 RMB (%)	11	3 (27.27)	4 (36.36)	4 (36.36)	9 (81.82)	1 (9.09)	1 (9.09)	4 (36.36)	1 (9.09)	6 (54.55)
	>3,000 RMB (%)	8	3 (37.50)	3 (37.50)	2 (25.00)	7 (87.50)	1 (12.50)	0 (0.00)	4 (50.00)	0 (0.00)	4 (50.00)
Participating sports consumption	≤500 RMB (%)	353	113 (32.01)	173 (49.01)	67 (18.98)	284 (80.45)	44 (12.46)	25 (7.08)	113 (32.01)	55 (15.58)	185 (52.41)
	501 ~ 1,000 RMB (%)	35	8 (22.86)	21 (60.00)	6 (17.14)	26 (74.29)	6 (17.14)	3 (8.57)	10 (28.57)	8 (22.86)	17 (48.57)
	1,001 ~ 2000 RMB (%)	5	2 (40.00)	1 (20.00)	2 (40.00)	4 (80.00)	1 (20.00)	0 (0.00)	2 (40.00)	1 (20.00)	2 (40.00)
	2001 ~ 3,000 RMB (%)	1	0 (0.00)	0 (0.00)	1 (100.00)	1 (100.00)	0 (0.00)	0 (0.00)	0 (0.00)	0 (0.00)	1 (100)
	>3,000 RMB (%)	1	1 (100.00)	0 (0.00)	0 (0.00)	1 (100.00)	0 (0.00)	0 (0.00)	1 (100)	0 (0.00)	0 (0.00)
Consumption of exercise rehabilitation services	≤500 RMB (%)	322	102 (31.68)	158 (49.07)	62 (19.25)	261 (81.06)	37 (11.49)	24 (7.45)	98 (30.43)	54 (16.77)	170 (52.80)
	501 ~ 1,000 RMB (%)	46	14 (30.43)	25 (54.35)	7 (15.22)	33 (71.74)	9 (19.57)	4 (8.70)	18 (39.13)	7 (15.22)	21 (45.65)
	1,001 ~ 2000 RMB (%)	13	4 (30.77)	5 (38.46)	4 (30.77)	11 (84.62)	2 (15.38)	0 (0.00)	4 (30.77)	3 (23.08)	6 (46.15)
	2001 ~ 3,000 RMB (%)	3	1 (33.33)	0 (0.00)	2 (66.67)	2 (66.67)	1 (33.33)	0 (0.00)	1 (33.33)	0 (0.00)	2 (66.67)
	>3,000 RMB (%)	11	3 (27.27)	7 (63.64)	1 (9.09)	9 (81.82)	2 (18.18)	0 (0.00)	5 (45.45)	0 (0.00)	6 (54.55)

Low: no significant change in breathing or heart rate; Moderate: slightly faster breathing and light sweating; High: rapid breathing, noticeably increased heart rate, and heavy sweating.

Sport consumption levels exhibited variation across different exercise frequency groups. For sports product consumption, individuals exercising 3–7 times per week accounted for the highest proportion across all spending categories, with 50.21% in the ≤500 RMB group and over half of the individuals in the 501–2000 RMB ranges. Respondents who exercised more than 7 times per week showed a higher tendency toward moderate or high consumption (e.g., 36.36% in the 2001–3,000 RMB range). A similar pattern was observed for participating sports and rehabilitation services: the 3–7 times/week group dominated all consumption levels, suggesting that regular exercise engagement is positively correlated with sport consumption. In particular, all respondents who spent more than 3,000 RMB on participating sports reported exercising at least once a week.

Across all three categories of sport consumption, individuals who exercised for ≤60 min per session constituted the majority of respondents, especially in the lowest spending bracket. For instance, 79.50% of those spending ≤500 RMB on sports products and 80.45% for participation fell into this group. However, those with longer exercise durations (61–120 min or >120 min) showed progressively higher tendencies toward mid-to-high consumption. For example, 21.4% of individuals who spent 1,001–2000 RMB on sports products exercised for more than 60 min. Similarly, in the exercise rehabilitation category, 19.57% of those spending 501–1,000 RMB reported exercising for 61–120 min. This trend suggests that increased duration of physical activity per session may foster higher levels of sports-related expenditures.

Perceived intensity was also associated with differences in consumption behavior. In general, individuals reporting moderate or high exercise intensity were more likely to fall into higher spending brackets. For example, 52.30% of those spending ≤500 RMB on sports products perceived their exercise as high intensity, and 50.00% of those spending >3,000 RMB perceived their activity as high intensity. Similarly, in the rehabilitation service category, 54.55% of respondents spending >3,000 RMB reported high-intensity activity. Notably, low-intensity exercisers were heavily concentrated in the lowest spending categories across all types, indicating a strong positive correlation between perceived exertion and sport consumption behavior.

Overall, individuals with higher frequency, longer duration, and greater intensity of exercise were more likely to engage in higher levels of sport consumption. This trend was particularly evident in spending on exercise rehabilitation services, which showed marked differences across exercise behavior groups. These findings highlight the behavioral link between exercise engagement and consumer investment in sport-related goods and services.

## Discussion

This study examined the associations between different types of sport consumption expenditures and physical activity behaviors among older adults in Wuhan, China. By categorizing sport-related spending into three domains: sports products, participatory services, and exercise rehabilitation, this study provides empirical evidence on the role of economic investment in shaping active aging trajectories.

The findings indicate that higher expenditures on sports consumption, particularly in the areas of sports products and exercise rehabilitation services, are significantly linked to increased physical activity frequency, longer session duration, and higher self-reported exercise intensity. These associations suggest that sport consumption may serve as a tangible manifestation of health-oriented investment behavior among older adults. The notion of sport-related spending as a form of “preventive health capital” is consistent with previous studies highlighting the positive influence of health-related consumption on physical activity participation (26, 27). Expenditures on items such as fitness gear, gym memberships, or rehabilitative services likely reflect not only access to resources but also a proactive health orientation, reinforcing the idea that material investments can lower participation barriers and facilitate behavioral change (28).

Socioeconomic disparities also emerged as influential factors in shaping both sport consumption patterns and physical activity behaviors (29, 30). Individuals with higher income and education levels were more likely to spend in the mid-to-high range across all sport consumption categories, corroborating existing literature that emphasizes the role of socioeconomic status in promoting healthier lifestyles among older adults (31, 32). This may be due to enhanced financial capacity, greater health literacy, or increased access to supportive environments that promote active living (33). The interplay between economic capital and cognitive resources underscores the importance of integrated public health strategies that address both material and informational barriers to active aging.

Despite these positive associations, a notable proportion of older adults reported limited financial engagement with sport-related goods and services. For instance, over 60% of participants spent no more than 500 RMB annually on sports products, and nearly 90% reported similarly low expenditures on participation services. This pattern aligns with prior findings indicating the critical role of publicly provided infrastructure, such as free parks, community exercise equipment, and volunteer-led fitness groups, in enabling physical activity among older adults with limited financial means (34–36). However, the relatively low uptake of exercise rehabilitation services suggests a potential gap between latent health needs and service utilization. Barriers such as cost, limited awareness, and skepticism regarding service efficacy may partially explain this underutilization (37, 38). Addressing these barriers is crucial, particularly in light of the rising prevalence of chronic conditions and functional decline in aging populations.

These findings have several implications for public health practice and aging-related policy development. Tailored strategies are necessary to support older adults with diverse socioeconomic profiles. For those with sufficient financial resources, expanding access to personalized and technologically integrated fitness options may enhance engagement (39–41). For individuals with constrained incomes, policy interventions such as subsidized programs, community-based offerings, and targeted vouchers may be essential to ensuring equitable participation. From a systems perspective, integrating sport consumption policy into broader frameworks for inclusive and age-friendly health promotion is essential for narrowing the participation gap and reducing health inequities.

Furthermore, the multidimensional nature of sport consumption, encompassing not just physical products, but also experiential services and therapeutic interventions, warrants greater recognition in both research and policy. Promoting healthy aging requires not only encouraging physical activity behavior itself, but also fostering supportive consumption environments through coordinated efforts across the public health, sports, and social sectors (42, 43).

Educational outreach also remains a critical lever for intervention. Older adults with lower levels of educational attainment may lack sufficient awareness of the health benefits associated with sport-related activities and services. Previous studies have shown that health literacy significantly mediates the relationship between socioeconomic status and physical activity engagement (44, 45).

Thus, culturally appropriate and accessible health education campaigns, particularly those utilizing peer-led or community-based delivery models, may be effective in enhancing participation rates among disadvantaged groups.

## Conclusion

This study provides empirical evidence that sports consumption expenditure is positively associated with physical activity behaviors among older adults in urban China. Specifically, expenditures on sports products, participatory activities, and exercise rehabilitation services are linked to higher frequency, longer duration, and greater intensity of physical activity. These findings suggest that sport-related spending is not merely a reflection of economic activity, but also an important behavioral and motivational factor that supports active aging. Our results highlight the need for differentiated public health strategies that consider older adults’ financial capacities, educational backgrounds, and access to sport-related services. While some older individuals are able and willing to invest in their physical well-being through sport consumption, a large proportion remain underserved due to limited resources or awareness. Policymakers should promote inclusive and equitable access to sport and fitness opportunities by expanding public facilities, subsidizing participation costs, and enhancing health education targeted at less advantaged groups. As population aging accelerates globally, understanding the drivers of healthy aging is increasingly urgent. This study underscores the potential of sports consumption, especially when embedded within supportive environments and policy frameworks, as a lever to promote physical activity, enhance well-being, and reduce health disparities in later life.

This study has several limitations that should be acknowledged. First, the cross-sectional design precludes causal inference regarding the directionality of the relationship between sports consumption and physical activity. Second, all key variables were measured using self-reported questionnaires, which may be subject to recall bias and social desirability bias; respondents may have over- or under-estimated their annual sports-related expenditures or typical exercise duration. Third, although several sociodemographic variables were included, important potential confounders, such as detailed health conditions (e.g., specific chronic diseases, functional limitations) and the accessibility and quality of community sports facilities, were not captured and may influence both consumption behaviors and activity patterns. Finally, our analysis focused only on contemporaneous associations and did not examine long-term or dynamic changes. Future longitudinal and intervention studies are needed to track changes in both spending and activity over time and to clarify the causal pathways underlying these associations.

## Data Availability

The original contributions presented in the study are included in the article/Supplementary material, further inquiries can be directed to the corresponding author.
