# Supplementary material for: Associations between physical activity participation and different types of sports consumption expenditures among older adults: a cross-sectional study
Source: Front Public Health. 2025 Dec 17;13:1713320. doi: 10.3389/fpubh.2025.1713320 (PMC12753444; doi:10.3389/fpubh.2025.1713320)
Supplement: Supplementary file 1 [file Table_1.doc]

**Questionnaire: Sociodemographic Information, Physical Activity Participation, and Sport Consumption Expenditure among Older Adults**

Dear Participant,

This questionnaire is intended for individuals aged 60 and above. It aims to understand your physical activity habits and spending related to sports and health over the past 12 months. The information you provide will be kept strictly confidential and used only for academic research. Your honest responses will help us better understand the needs of older adults and improve public health and sports services.

Please read each question carefully and choose the answer that best reflects your situation. There are no right or wrong answers.

**Section 1: Sociodemographic Information**

**1. Sex**

(0) Male

(1) Female

**2. Education Level**

(1) Primary school or below

(2) Junior high school

(3) High school / Technical / Vocational school

(4) Bachelor’s degree or above

**3. Monthly Personal Income (before tax)**

(1) ≤ 4000 RMB

(2) 4001–6000 RMB

(3) > 6000 RMB

**4. Annual Household Income**

(1) ≤ 40,000 RMB

(2) 40,001–80,000 RMB

(3) > 80,000 RMB

**Section 2: Physical Activity Participation**

**5. How often do you do physical fitness activities each week?**

(1) 1–2 times per week

(2) 3–7 times per week

(3) More than 7 times per week

**6. How long does each exercise session usually last?**

(1) Less than or equal to 60 minutes

(2) 61–120 minutes

(3) More than 120 minutes

**7. How would you describe the intensity of your regular exercise?**

(1) Low – no obvious change in breathing or heart rate

(2) Moderate – breathing slightly faster, some light sweating

(3) High – heavy sweating, fast breathing, and fast heart rate

**Section 3: Sport Consumption Expenditure in the Past Year**

**8. How much did you spend on sports products (e.g., sportswear, shoes, hats, equipment)?**

(1) ≤ 500 RMB

(2) 501–1000 RMB

(3) 1001–2000 RMB

(4) 2001–3000 RMB

(5) > 3000 RMB

**9. How much did you spend on participating in sports (e.g., coaching, gym/club memberships, venue rentals, ticket purchases)?**

(1) ≤ 500 RMB

(2) 501–1000 RMB

(3) 1001–2000 RMB

(4) 2001–3000 RMB

(5) > 3000 RMB

**10. How much did you spend on exercise rehabilitation (e.g., exercise prescriptions, physical rehabilitation services, nutritional supplements)?**

(1) ≤ 500 RMB

(2) 501–1000 RMB

(3) 1001–2000 RMB

(4) 2001–3000 RMB

(5) > 3000 RMB
